# Job Crafting as the Missing Link: Understanding Its Role in Nurses’ Work Engagement

**DOI:** 10.1155/jonm/9420686

**Published:** 2025-12-28

**Authors:** Kyungjin Lee, Ja Kyung Seo, Seung Eun Lee

**Affiliations:** ^1^ Severance Hospital, Seoul, 03722, Republic of Korea, yuhs.or.kr; ^2^ Department of Psychology, Psychological Science Innovation Institute, Yonsei University, Seoul, 03722, Republic of Korea, yonsei.ac.kr; ^3^ College of Nursing, Mo-Im KIM Nursing Research Institute, Yonsei University, Seoul, 03722, Republic of Korea, yonsei.ac.kr

## Abstract

**Aim:**

This study examined how nurses’ positive psychological capital and positive work environment influence work engagement and investigated the mediating role of job crafting in these relationships.

**Background:**

Nurses’ work engagement is essential for achieving hospital goals, as it directly impacts patient care quality and organizational performance. While both personal resources such as positive psychological capital and job resources such as a positive work environment influence work engagement, the mechanisms underlying these relationships remain unclear. This study focuses on job crafting as a key mechanism linking these factors to work engagement.

**Methods:**

This descriptive correlational study analyzed data from 243 registered nurses working in general hospitals in South Korea. A path analysis was used to test a hypothesized model.

**Results:**

Both positive psychological capital (*β* = 0.376, *p* < 0.001) and a positive work environment (*β* = 0.279, *p* < 0.001) had significant positive effects on nurses’ work engagement. Job crafting partially mediated the relationship between positive psychological capital and work engagement (*β* = 0.147, 95% confidence interval (CI) = [0.086, 0.221]) as well as the relationship between a positive work environment and work engagement (*β* = 0.029, 95% CI = [0.004, 0.070]).

**Conclusion:**

Nurses with higher positive psychological capital and those working in a positive work environment are more likely to be involved in job crafting, which in turn enhances their work engagement. These findings suggest that both individual and organizational strategies are essential in promoting individual nurses’ job crafting and work engagement.

**Implications for Nursing Management:**

Nursing management should cultivate a positive work environment and provide opportunities for professional growth to improve nurses’ job crafting and work engagement. Strategies such as adequate staffing, managerial support, and autonomy in decision‐making can help sustain engagement, ultimately improving patient care and organizational performance.

## 1. Introduction

Work engagement is a positive psychological state [1] that enhances employee motivation and commitment to their work and improves organizational performance [2]. Highly engaged employees are enthusiastic about their roles and have a strong sense of belonging, which increases their productivity and efficiency [2]. In nursing, work engagement holds particular importance because of its association with increased retention intention [3], greater work commitment [4], higher quality of patient care [5], and improved patient safety outcomes [6]. Engaged nurses are more adaptable and employ diverse methodological approaches, which enhances their overall standard of care [7]. Given these significant implications, identifying the factors that promote nurses’ work engagement is essential [8].

To identify the antecedents of work engagement, we draw on the job demands–resources (JD–R) model [9], which proposes that work engagement arises when personal and job resources enable them to manage job demands and foster personal growth [10]. In this model, positive psychological capital—a multifaceted concept encompassing hope, self‐efficacy, resilience, and optimism—is recognized as a key personal resource [11, 12]. Individuals with high psychological capital tend to maintain an optimistic outlook and a strong belief in their ability to overcome challenges, which helps them stay motivated [13] and fosters greater engagement with their work [14]. Prior research has shown a positive relationship between psychological capital and nurses’ work engagement [15].

**Hypothesis 1.**

Positive psychological capital is positively associated with work engagement.

Within the JD–R model [9], a positive work environment functions as a key job resource that facilitates employee engagement. In the nursing context, this environment is typically characterized by managerial support, collaborative nurse–physician relationships, foundations of nursing for quality care, adequate staffing, and involvement in decision‐making [16]. When nurses feel supported and have access to job resources, they are likely to develop intrinsic motivation, which fosters greater work engagement [17]. Longitudinal evidence suggests that the presence of job resources in positive work environments helps sustain increases in engagement over time [18]. Empirical research has also shown that nurses who perceive their work environment favorably report greater engagement with their work [19].

**Hypothesis 2.**

A positive work environment is positively associated with work engagement.

In addition to fostering work engagement, personal and job resources can stimulate nurses’ proactive behaviors. One such behavior is job crafting, defined as employees’ self‐initiated modifications to their job tasks, relationships, or cognitive perceptions to optimize alignment with their competencies and preferences [20]. Individuals with higher levels of positive psychological capital are more likely to engage in job crafting, as their self‐efficacy, optimism, and resilience enable them to reshape their roles and navigate challenges [21]. Additionally, when employees perceive their environment as positive, they are more likely to modify aspects of their jobs with confidence in their abilities [22]. Since job crafting involves actively managing job demands and resources [20], both personal and job resources serve as key enablers of this proactive behavior.

**Hypothesis 3.**

Positive psychological capital and a positive work environment are positively associated with job crafting among nurses.

Job crafting enables individuals to actively modify their work roles and environments, thereby fostering greater work engagement [23, 24]. Through this process, employees often develop a more positive perception of their work and attribute greater value to it, leading to increased work motivation [25]. Those who actively engage in job crafting are more likely to find meaning in their work, which in turn enhances their commitment and engagement [26]. By proactively reshaping their roles, optimizing their work environments, and reframing their perceptions of work, nurses can further strengthen work engagement [27].

**Hypothesis 4.**

Job crafting is positively associated with work engagement.

Personal and job resources are central to work engagement, with job crafting as a key behavioral pathway linking them. In the extended JD–R model [28], employees draw on these resources to proactively adjust their task boundaries, work methods, and relationships, which strengthens motivation and engagement. Individuals with higher optimism and self‐efficacy are more likely to take the initiative and embrace challenges at work [29], making them more inclined to engage in job crafting and, in turn, to experience greater work engagement [30]. Likewise, work environments that support autonomy and provide adequate resources enable such adjustments, thereby strengthening work engagement [20]. Building on this mechanism, prior studies indicate that each type of resource can contribute to engagement by facilitating job crafting. For instance, Park and Ha [31] showed that positive psychological capital indirectly influences nurses’ work engagement through job crafting, and Jiang et al. [32] indicated that job resources positively influence work engagement through job crafting among tobacco retailers. While prior nursing research has examined personal (self‐efficacy) and job (peer support) resources together via job crafting [33], the specific pairing of positive psychological capital and a positive work environment and whether they operate in parallel through the same crafting mechanism has not been examined. Accordingly, we propose a parallel mediation model in which both positive psychological capital (personal resource) and a positive work environment (job resource) promote job crafting, which in turn enhances nurses’ work engagement.

**Hypothesis 5.**

Job crafting mediates the relationships between positive psychological capital and work engagement as well as between a positive work environment and work engagement.

## 2. Materials and Methods

### 2.1. Setting and Sample

This study employed a correlational, cross‐sectional design. Data were collected from an online survey in April and May 2024. Survey links were distributed through the nursing departments of three general hospitals in South Korea. Designated research coordinators at each hospital shared the survey with eligible nurses through internal communication channels. The inclusion criteria for participation were direct care nurses who (1) had worked at their current hospital for at least 1 year, (2) were permanent staff nurses (i.e., not temporary or contract‐based), and (3) understood the purpose of the study and voluntarily agreed to participate. Nurses who worked as managers and nurses who were not directly involved in patient care (e.g., those in administrative, educational, or research roles) were excluded. Interested nurses could access the survey link, review the study’s purpose and procedures, and provide informed consent electronically before beginning the questionnaire.

A total of 252 responses were collected. Of those, nine were excluded due to indicators of poor data quality (e.g., identical responses across items; inconsistencies between reverse‐coded and nonreverse‐coded items). Hence, 243 valid responses were used in the final analysis. Structural equation modeling and a path analysis generally require a minimum sample size of 200 to produce stable and reliable estimates, and adequacy should be judged by the case‐to‐parameter ratio [34]. Given our model (two independents, one mediator, one dependent, and two controls), 243 cases produce a ratio well above 10:1, indicating an adequate sample size.

### 2.2. Measures

#### 2.2.1. Outcome Variable

*Work engagement* was assessed using the Korean version of the Utrecht Work Engagement Scale (nine items), which has demonstrated adequate validity and reliability with a Korean sample [35]. Responses were rated on a seven‐point Likert scale ranging from 0 (*never*) to 6 (*always*), with a higher mean score indicating greater work engagement. The Cronbach’s alpha of the scale was 0.85 in a previous Korean study [36], and in this study, the alpha was 0.91.

#### 2.2.2. Predictor Variables

*Positive psychological capital* was measured using the Korean version of the Psychological Capital Questionnaire (24 items) provided by Mind Garden (http://www.mindgarden.com), a U.S. company that holds the copyright for the measure. This instrument comprises four subscales (hope, optimism, self‐efficacy, and resilience), each containing six items [37]. Responses were rated on a six‐point Likert scale ranging from 1 (*strongly disagree*) to 6 (*strongly agree*), with a higher mean score indicating higher positive psychological capital. The Cronbach’s alpha of the scale was 0.93 in a previous Korean study [31], and in this study, the alpha was 0.92.

A *positive work environment* was assessed using the Korean version of the Practice Environment Scale of Nursing Work Index (29 items), which has demonstrated adequate validity and reliability among Korean nurses [16]. Responses were rated on a four‐point Likert scale ranging from 1 (*strongly disagree*) to 4 (*strongly agree*), with a higher mean score indicating a more favorable nursing work environment. The Cronbach’s alpha of the scale was 0.93 in a previous Korean study [16], and in this study, the alpha was 0.92.

#### 2.2.3. Mediator Variable

*Job crafting* was measured using the Korean version of the Job Crafting Questionnaire (15 items), which has shown adequate reliability and validity with a Korean sample [25]. Responses were rated on a six‐point Likert scale ranging from 1 (*strongly disagree*) to 6 (*strongly agree*), with a higher mean score indicating a higher level of job crafting. The Cronbach’s alpha of the scale was 0.92 in a previous Korean study [31], and in this study, the alpha was 0.87.

#### 2.2.4. Demographic Characteristics

The demographic characteristics included age (in years), sex (male/female), education level (associate, bachelor’s, master’s or higher), total years of nursing experience, and current work unit (general vs. specialty unit).

### 2.3. Data Analysis

Descriptive statistics and Pearson’s correlation coefficients were calculated using SPSS version 27.0 to understand the demographic characteristics of the participants, examine the key variables, and assess the relationships among the key variables. A path analysis was performed using AMOS version 26.0 to test the hypothesized relationships among the variables. We controlled for age and work unit in the path analysis because these factors influence work engagement [38, 39].

The path analysis was performed using maximum likelihood estimation, with the model fit assessed based on established benchmarks: the comparative fit index (CFI > 0.95), the Tucker–Lewis index (TLI > 0.95), and the root mean square error of approximation (RMSEA < 0.06) [40]. A bootstrapping procedure with 5000 resamples was employed to examine the significance of the direct and indirect effects, producing 95% bias‐corrected confidence intervals (CIs) [41].

### 2.4. Ethical Considerations

This study received approval from the Institutional Review Board at Yonsei University Health Systems (#4‐2024‐0256). Before participation, all the individuals provided voluntary informed consent. Before taking part in the study, the participants received detailed information on its purpose, the methodology, and their rights, including assurances of anonymity, the right to withdraw at any time without consequences, and the restricted use of their data solely for research purposes.

## 3. Results

### 3.1. Participants’ Characteristics

Table 1 summarizes the characteristics of the participants. Their average age was 31.36 years (*SD* = 5.99), and their mean nursing experience was 7.90 years (*SD* = 5.86). Most participants were female (88.1%) and held a bachelor’s degree (88.5%). In terms of work unit, nearly two‐thirds were employed in specialty units, while the remainder worked in general units.

**Table 1:** Characteristics of the participants (*N* = 243).

Characteristic	Category	*n* (%)	*M* (*SD*)
Age (years)			31.36 (5.99)
Nursing experience (years)			7.90 (5.86)
Sex	Male	29 (11.9)	
	Female	214 (88.1)	
Education level	Associate degree	9 (3.7)	
	Bachelor’s degree	215 (88.5)	
	Master’s or higher	19 (7.8)	
Work unit	General unit	84 (34.6)	
	Specialty unit^a^	159 (65.4)	

^a^Specialty unit included the intensive care unit, emergency department, operating room, and others.

Abbreviations: *M*, mean; *SD*, standard deviation.

### 3.2. Descriptive Statistics and Correlation Analysis

The data met the assumptions of normality, as the skewness values ranged from −0.233 to −0.022 and the kurtosis values ranged from −0.207 to 0.906, well within the acceptable thresholds of 3.0 and 10.0, respectively [34]. The variance inflation factors of the mediators and predictors ranged from 1.021 to 1.873, suggesting no concerns about multicollinearity [34].

As shown in Table 2, positive psychological capital demonstrated a significant correlation with a positive work environment (*r* = 0.422, *p* < 0.001), job crafting (*r* = 0.650, *p* < 0.001), and work engagement (*r* = 0.648, *p* < 0.001). A positive work environment was significantly related to both job crafting (*r* = 0.372, *p* < 0.001) and work engagement (*r* = 0.526, *p* < 0.001). Similarly, job crafting was significantly and positively related to work engagement (*r* = 0.599, *p* < 0.001).

**Table 2:** Means, standard deviations, and correlations of the variables.

Variable	1	2	3	4
1. Positive psychological capital	—			
2. Positive work environment	0.422^∗∗∗^	—		
3. Job crafting	0.650^∗∗∗^	0.372^∗∗∗^	—	
4. Work engagement	0.648^∗∗∗^	0.526^∗∗∗^	0.599^∗∗∗^	—
*M*	4.01	2.42	4.04	2.96
*SD*	0.56	0.44	0.67	0.91

*Note:* All the scales are presented as mean values. Ranges: 0–6 for work engagement, 1–6 for psychological capital and job crafting, and 1–4 for a positive work environment.

Abbreviations: *M*, mean; *SD*, standard deviation.

^∗∗∗^ *p* < 0.001.

### 3.3. Path Analysis

**Figure 1:**
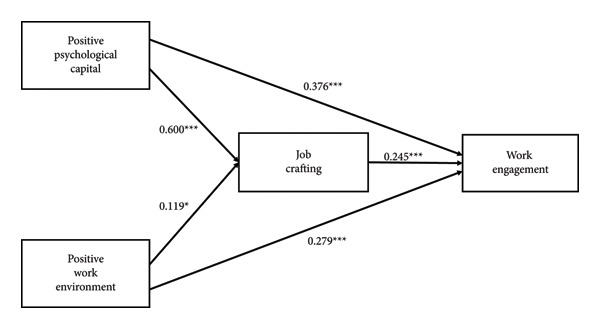
Path analysis results of the hypothesized mediation model with standardized coefficient estimates. ^∗^ *p* < 0.05, ^∗∗∗^ *p* < 0.001.

A path analysis was conducted to examine the relationships among positive psychological capital, a positive work environment, job crafting, and work engagement. The model fit indices indicated a satisfactory level, with *χ* ^2^ (2) = 2.484, CFI = 0.999, TLI = 0.999 (both > 0.95), and RMSEA = 0.032 (< 0.06), supporting the adequacy of the model. As shown in Figure 1, positive psychological capital demonstrated a significant direct effect on job crafting (*β* = 0.600, *p* < 0.001) and work engagement (*β* = 0.376, *p* < 0.001). A positive work environment also had a significant direct effect on both job crafting (*β* = 0.119, *p* < 0.05) and work engagement (*β* = 0.279, *p* < 0.001). Additionally, job crafting exhibited a significant positive effect on work engagement (*β* = 0.245, *p* < 0.001), indicating its mediating role in the model.

Furthermore, as shown in Table 3, positive psychological capital and a positive work environment had significant indirect effects on work engagement through job crafting. Job crafting significantly mediated the relationship between positive psychological capital and work engagement, as the 95% CI did not include zero (*β* = 0.147, 95% CI = [0.086, 0.221]). Similarly, job crafting significantly mediated the association between a positive work environment and work engagement (*β* = 0.029, 95% CI = [0.004, 0.070]), with the CI also excluding zero. These findings support the role of job crafting as a key mechanism linking positive psychological capital and a positive work environment to work engagement.

**Table 3:** Standardized indirect effects in the hypothetical model.

Path	*B*	*SE*	*P*	95% CI (lower, upper)
Direct effects				
Positive psychological capital ⟶ Work engagement	0.376	0.059	< 0.001	(0.259, 0.491)
Positive work environment ⟶ Work engagement	0.279	0.049	0.001	(0.183, 0.371)
Indirect effects				
Positive psychological capital ⟶ Job crafting ⟶ Work engagement	0.147	0.034	< 0.001	(0.086, 0.221)
Positive work environment ⟶ Job crafting ⟶ Work engagement	0.029	0.017	0.019	(0.004, 0.070)

Abbreviations: CI, bias‐corrected confidence interval; *SE*, standard error.

## 4. Discussion

This study investigated the associations among positive psychological capital, a positive work environment, job crafting, and work engagement, focusing on the mediating role of job crafting. The results provide valuable insights into how these factors enhance work engagement among nurses.

Consistent with previous studies [8, 42], our findings showed that positive psychological capital is positively associated with work engagement, supporting Hypothesis 1. Nurses with higher positive psychological capital approach workplace challenges with optimism, perceiving difficulties as opportunities for growth and solutions as attainable [42]. As a personal resource, psychological capital is associated with greater resilience, which helps sustain task focus under stressful conditions [14]. In addition, such individuals cope more effectively with job demands, managing pressures without substantial loss of motivation [43]. Taken together, this evidence supports positive psychological capital as a key personal resource that helps sustain nurses’ work engagement [44].

Our data showed that a positive work environment was related to higher work engagement, supporting Hypothesis 2 and echoing earlier findings [45]. Within the JD–R framework [9], a positive work environment functions as a job resource that facilitates work engagement [46]. It expands opportunities for achievement and recognition, which are experiences linked to stronger engagement [47]. When nurses feel supported, they are more likely to internalize the value of their work and adopt favorable attitudes toward their roles and the organization [48], thereby fostering greater enthusiasm and commitment [49]. Moreover, positive work environments help fulfill nurses’ psychological needs (e.g., competence and relatedness), further strengthening engagement [50]. Collectively, these findings underscore the critical role of a positive work environment in promoting nurses’ work engagement.

Furthermore, both positive psychological capital and a positive work environment were positively associated with job crafting, supporting Hypothesis 3. Nurses who feel confident in their abilities and optimistic about overcoming challenges are more inclined to take initiative—for example, redefining task boundaries, adopting more effective methods, and reshaping work relationships—key forms of job crafting [51, 52]. Regarding the work environment, sufficient staffing, fair compensation, and involvement in decision‐making provide conditions that enable and encourage such behavior [53]. Adequate resources and strong managerial support are likewise essential; without them, employees encounter barriers to modifying roles and responsibilities [54]. Taken together, these findings suggest that job crafting is not merely an individual proactive behavior but is shaped by both psychological and organizational factors that enable nurses to adapt their work roles effectively.

Consistent with previous research [55], job crafting was positively related to work engagement, supporting Hypothesis 4. Employees who redefine task boundaries, reframe how they approach their work, and cultivate supportive relationships—core forms of job crafting—tend to experience their jobs as more meaningful and manageable [26]. These changes may enhance perceived control and competence and generate positive affect, processes associated with higher engagement [56, 57]. Collectively, these patterns position job crafting as a proximal behavioral pathway to greater engagement.

Supporting Hypothesis 5, job crafting partially mediated the associations of psychological capital and a positive work environment with work engagement when both predictors were entered simultaneously. This pattern is broadly consistent with prior work showing that personal (e.g., grit and self‐efficacy) and job resources (e.g., feedback, autonomy, supervisor support, and career opportunities) relate to engagement via job crafting [33, 58, 59]. Our study complements this literature by jointly estimating both resources and showing their indirect effects after accounting for shared variance. Taken together, the results suggest that job crafting operates as a common pathway from personal and job resources to engagement, with effects that are concurrent and partial, rather than exhaustive.

### 4.1. Implications for Nursing Management

Enhancing nurses’ work engagement requires coordinated efforts at the individual and organizational levels, aligned with our finding that job crafting serves as a key link between personal and job resources to engagement. At the individual level, nurses can pursue self‐directed learning and proactive problem‐solving to build psychological capital [60], which in our model was more strongly associated with job crafting than work environment. Nursing management can support this by implementing structured psychological capital strategies (e.g., positive reframing, goal‐setting) [61] and by creating conditions that enable job crafting and improve work engagement, such as timely feedback, greater autonomy, and development opportunities that enhance meaning and motivation [17, 62]. At the organizational level, a positive work environment—with adequate staffing, support for continuous professional development, and shared governance that involves nurses in unit‐level decisions (e.g., patient care activities, scheduling)—remains an important enabler of autonomy and engagement [45, 50, 55]. Reflecting our parallel‐mediation result, these actions emphasize parallel attention to both personal and job resources to activate job crafting, rather than addressing either in isolation. Together, these multilevel actions can strengthen both personal resources and job resources while creating space for job crafting, thereby reinforcing nurses’ work engagement.

### 4.2. Limitations of the Study

This study has some limitations. One key limitation is its cross‐sectional design, which makes it difficult to determine the causal relationships among the study variables. While the hypothesized model was based on a theoretical framework and supported by prior research, we cannot rule out the possibility of reciprocal or alternative causal pathways. Another limitation is the reliance on self‐reported data, which may introduce bias such as common method bias and social desirability bias. The participants may have responded in ways they perceived as socially appropriate rather than reflecting their actual behaviors. Additionally, the generalizability of the findings is limited by the use of convenience sampling. The sample, drawn from a specific group of nurses who were readily accessible, may not fully represent nurses in diverse healthcare settings or cultural contexts. Future research could incorporate more diverse samples across various healthcare institutions and regions to enhance external validity. Furthermore, employing longitudinal designs and mixed‐methods approaches could help address these limitations, reduce bias, and improve the robustness of the findings.

## 5. Conclusions

This study examined the relationships among positive psychological capital, a positive work environment, job crafting, and work engagement, identifying job crafting as a significant mediator in these relationships. Positive psychological capital and a positive work environment were both directly associated with greater work engagement and indirectly influenced engagement through job crafting. These findings align with the JD–R model [9], highlighting how both personal resources and job resources help foster proactive work behavior and heightened work engagement. By enhancing positive psychological capital, improving work environments, and encouraging job crafting, healthcare organizations can create conditions that sustain nurses’ work engagement and improve organizational outcomes.

## Disclosure

All the authors read and approved the final version of the manuscript.

## Conflicts of Interest

The authors declare no conflicts of interest.

## Author Contributions

K.L. and S.E.L. conceptualized and designed the study. K.L. collected and managed the data, performed the statistical analysis, and drafted the initial manuscript. J.K.S. reviewed and validated the results, providing revisions. S.E.L. supervised the study and made substantial contributions to the revision of the manuscript.

## Funding

No funding was received for this study.

## Data Availability

The data that support the findings of this study are available from the corresponding author upon reasonable request.
